# Supplementary material for: No relation between Body Mass Index and neurocognitive recovery in abstinent alcohol dependent patients?
Source: PLoS One. 2025 Oct 9;20(10):e0333790. doi: 10.1371/journal.pone.0333790 (PMC12510537; doi:10.1371/journal.pone.0333790)
Supplement: Attachment — Submitted filename: 20250723 response to reviewers_BMI paper.docx [file pone.0333790.s003.docx]

**POINT TO POINT REPLY**

Reviewer 1

1. *"Line - 209 "Normality was judged by visual inspection of the data. A skewness and kurtosis between -2 and +2 were accepted for normality." The fact that you calculated a number to compare to +/-2 suggestess more was done than visual inspection.*

**Authors reply:** *we clarified this by adding the following "In case of doubt, skewness and kurtosis were calculated to Z-scores (see for example [47]), where a score between -2 and +2 was accepted for normality [48]" (lines 227 – 229)*

2. *Line - 244 "All significant relations (p < 0.15)", You are using a non-standard alpha criterion. Add in the text a justification for not using 0.05.*

**Authors reply:** *we adjusted our explanation in line 238 to clarify this value. “*A more tolerant p-value (p < 0.15) is used to minimalize type II error. “ *(line 238)*

3. *Line- 400 "as the categorical BMI value", should be "and the categorical BMI value"*

**Authors reply:** *we have changed this as suggested.*

4. *Line - 443 " all-in-all", replace or remove this phrase"*

**Authors reply:** *we have changed this as suggested.*

Reviewer 2

No comments
